# A systematic review of spatial decision support systems in public health informatics supporting the identification of high risk areas for zoonotic disease outbreaks

**DOI:** 10.1186/s12942-018-0157-5

**Published:** 2018-10-30

**Authors:** Rachel Beard, Elizabeth Wentz, Matthew Scotch

**Affiliations:** 1 College of Health Solutions, Arizona State University, Phoenix, AZ USA; 2 Center for Environmental Health Engineering, Biodesign Institute, Arizona State University, Tempe, AZ USA; 3 School of Geographical Sciences and Urban Planning, Arizona State University, Tempe, AZ USA

**Keywords:** Spatial decision support systems, Public health informatics, Decision making, computer-assisted, Zoonoses

## Abstract

**Background:**

Zoonotic diseases account for a substantial portion of infectious disease outbreaks and burden on public health programs to maintain surveillance and preventative measures. Taking advantage of new modeling approaches and data sources have become necessary in an interconnected global community. To facilitate data collection, analysis, and decision-making, the number of spatial decision support systems reported in the last 10 years has increased. This systematic review aims to describe characteristics of spatial decision support systems developed to assist public health officials in the management of zoonotic disease outbreaks.

**Methods:**

A systematic search of the Google Scholar database was undertaken for published articles written between 2008 and 2018, with no language restriction. A manual search of titles and abstracts using Boolean logic and keyword search terms was undertaken using predefined inclusion and exclusion criteria. Data extraction included items such as spatial database management, visualizations, and report generation.

**Results:**

For this review we screened 34 full text articles. Design and reporting quality were assessed, resulting in a final set of 12 articles which were evaluated on proposed interventions and identifying characteristics were described. Multisource data integration, and user centered design were inconsistently applied, though indicated diverse utilization of modeling techniques.

**Conclusions:**

The characteristics, data sources, development and modeling techniques implemented in the design of recent SDSS that target zoonotic disease outbreak were described. There are still many challenges to address during the design process to effectively utilize the value of emerging data sources and modeling methods. In the future, development should adhere to comparable standards for functionality and system development such as user input for system requirements, and flexible interfaces to visualize data that exist on different scales.

PROSPERO registration number: CRD42018110466.

**Electronic supplementary material:**

The online version of this article (10.1186/s12942-018-0157-5) contains supplementary material, which is available to authorized users.

## Introduction

The current global population of 7.6 billion persons is expected to reach 9.8 billion by 2050 with an increasing number living in high density urban areas. The combination of high population density with increased global mobility of the human population potentially leads to growing exposure to dangerous zoonotic diseases. Technological advances, however, offer the opportunity to better understand patterns of disease spread, underlying conditions, and distribution of vulnerable populations. Spatial decision support tools can equip heath care officials with the data, analytics, information, modeling capacity, and visual tools to effectively make decisions and policy recommendations to improve public health outcomes. However, the literature shows that there have been missed opportunities, false starts, and gaps in the development of such tools. This paper identifies the strengths and challenges of spatial decision support system in public health informatics through a systematic literature review and offers insights on the significant advances, best practices, and gaps in knowledge.

## Background

Zoonotic diseases are a prominent concern in public health, with the rise or reemergence of disease-causing pathogens such as Middle Eastern Respiratory Syndrome (MERS), Ebola virus, Zika virus, West Nile virus (WNV), and numerous influenza strains across the globe [1, 2]. Transference from animal reservoirs to the human population is a concern among populations of mingling and mobile species across various geographic scales [3, 4]. Approximately 60% of all human pathogens that negatively impact overall population health are derived from animals and a new emerging disease manifests approximately every 8 months [5, 6]. With each new introduction, it has been found that 60–80% of documented emerging infectious diseases originated in animals [7].

In the United States, the federal government and individual states conduct surveillance of infectious diseases but are limited in the scope of their analyses due to available data, resources and training, which is intensified by the growing development and application of informatics techniques. This development includes the introduction of digital disease surveillance systems such as HealthMap [8] which provides visualization of current disease outbreaks detected through data aggregation of online data sources such as ProMED [9], RSS feeds, Twitter and news reports using automated datamining [10]. Traditionally, surveillance of clusters of at risk areas has been passive, as agencies rely on case reporting by clinicians, laboratories, and the public. Reportable disease data constitutes a suspected or infected case and addresses: who, what, when, and where did the infection occur. Identification of high risk-areas or clusters of disease outbreak using digital data as a means of early warning and prevention is gaining traction as public health practitioners have compared local surveillance network performance with tools such as HealthMap in order to assess utility as a supplementary tool [11]. Other emerging sources of data by which outbreaks are assessed in this modern era are drawing on genetic data, primarily to identify viral strain types and to assess pathogenicity. These data enable public health practitioners such as epidemiologists to study the infectious agent itself, often using results from local laboratory strain typing and sequencing efforts [12]. Other fields such as molecular epidemiology, landscape epidemiology and phylogeography have demonstrated the value of incorporating infectious disease genetics and spatial analysis to address ad hoc population health research [13–17].

Recent literature indicates that public health officials have begun to address disease surveillance by incorporating spatial and temporal components of reportable disease data to model outbreaks and using geographic information systems (GIS) [18]. These methods include statistical and GIS software to produce disease maps using an array of data types such as clinical, the physical environment, or human mobility data to identify outbreaks or disease clustering. However, here has been little focus on employing these approaches by local health departments (LHDs) for differentiating strains of circulating zoonotic viruses, though the opportunity to do so is growing with the increasing amount of genetic data being generated via Next Generation Sequencing (NGS), and public sequence databases such as GenBank and the Influenza Research Database (IRD) [19, 20].

While the application of GIS and spatial statistics has advanced visualization and decision-making capabilities for disease detection, similar technological advances have been introduced and merged into integrated systems in other informatics related fields called a spatial decision support systems (SDSS). SDSS emerged from the more general decision support systems (DSSs), used widely across public health, governance, and environmental management fields. For example, electronic health records (EHRs) can incorporate DSS to assist medical staff with a variety of tasks such as treatment plans or alerts of contraindications in medications [21]. SDSS are computer-based systems that allow decision makers to take advantage of available data to solve spatially related problems in a more dynamic and integrated interface that allows for data organization, analysis and visualization [22]. Implementation of such systems have the potential to facilitate public health decision makers with many tasks from detecting high risk locations for influenza outbreaks, or distribution of medical facilities, vaccines and staff based on the affected population distribution [23, 24]. While technology and analytical software has become more sophisticated, a movement to enable decision makers to more fully explore available data to develop actionable and evidence based planning has grown [25].

The term spatial decision support system was first introduced in a series of conference proceedings from 1983 to 1985 by Jerome Dobson, in addition to Hopkins and Armstrong respectively [26, 27]. There are a few examples of prototype SDSS projects that predate the term, such as IBM’s GADS (Geodata analysis and display system) which allowed users to analyze and display geographic data [28]. However, for many years SDSS remained in a developmental phase in which decision support systems and GIS techniques were integrated into a more cohesive framework. The 1990’s saw the rise of SDSS that were frequently built using Esri’s ArcGIS software, often to demonstrate proposed architectural designs. The first implementations of modern SDSS occurred in the early 2000’s and continue today, which take advantage of advances in the world wide web that allows for web-services, data warehousing and advanced analytical processing [29].

**Fig. 1:**
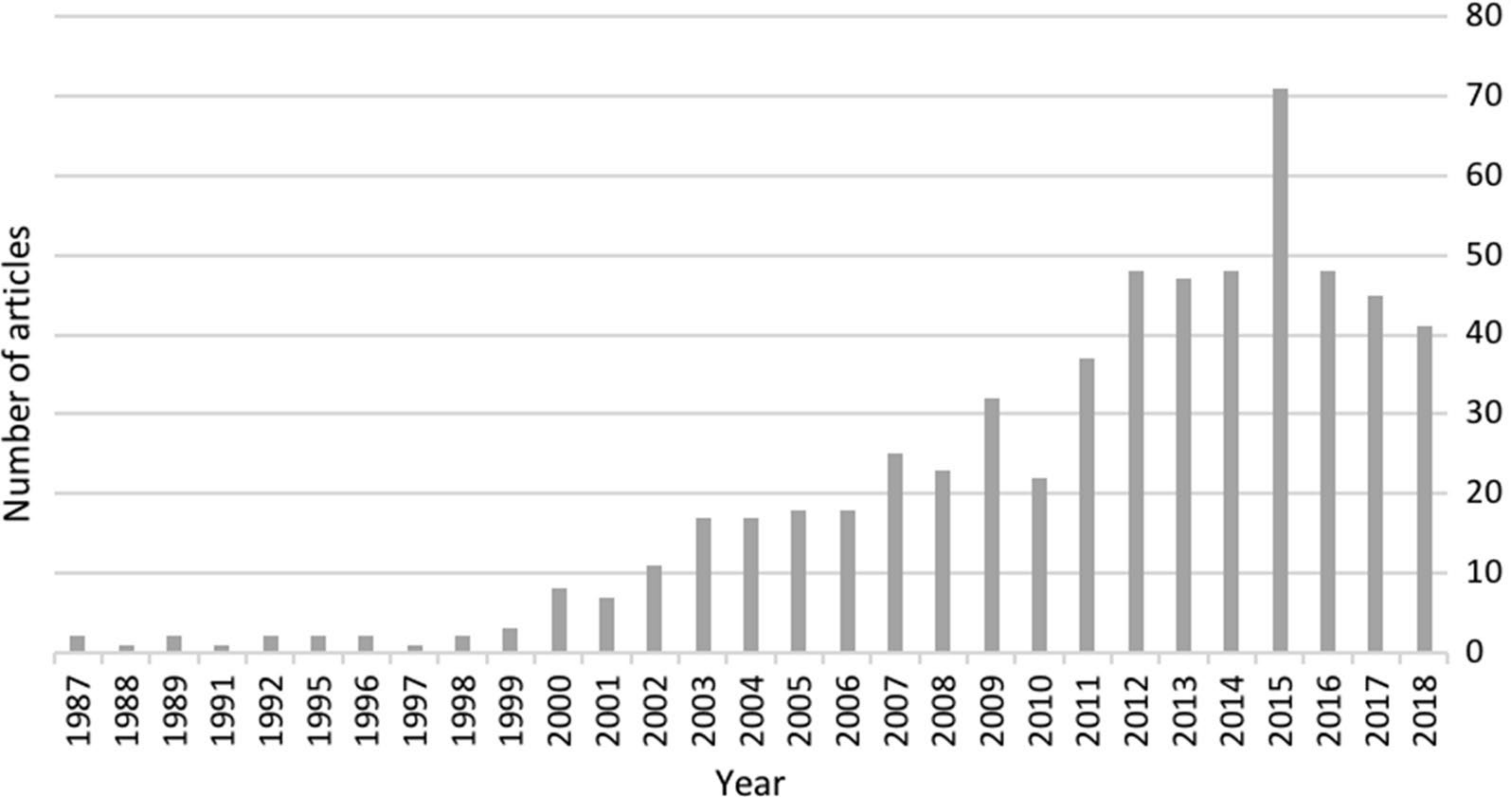
Number of papers that mention spatial decision support system by year in PubMed

Features that differentiate SDSS from other related systems, including clinical decision support tools, and GIS software are derived from the complex decision-making process all users engage in across multiple fields. Public health practitioners consider data such as reported cases, demographics, recent exposures, location and many other aspects while managing potential and current disease outbreaks. Due to scenarios of this complex nature, spatial problems have been referred to as “semi-structured” because they cannot be fully articulated, nor are the procedures carried out in a decision making process consistent from one investigation to the next [30]. Often decision-making software focuses on developing tools for a pre-defined decision-making process, which are often too constricting. Instead, early in the introduction of SDSS, Densham [30] emphasized that highly adaptive tools within a problem solving environment are needed. Since this time, the characteristics of modern SDSS have been defined by Sugumaran and DeGroote [29]. These characteristics, which include the ability to perform spatial analysis, visualization, and multiple scenario evaluation all within the same system have increased in popularity in the past decade as indicated by published literature using the term in PubMed (Fig. 1).

Several example SDSS reported in the literature that are intended to aid public health decision makers in situations ranging from capacity analysis for local hospital beds to national preparedness for influenza outbreaks. Many recently developed systems web-based surveillance systems or platforms, though some are distributed as software packages. Functionality often includes geo-visualization of observed disease cases or outbreaks, to assisting in the actual decision to be made such as distribution of vaccination supplies [31, 32]. For example, Huang et al. [33] developed a SDSS for risk assessment of airline travel on global disease outbreaks. This tool was intended to aid the decision process, readily quantifying and comparing risk levels to assist end users with targeting priority routes in terms of prevention of spread, or mitigation efforts. In contrast, Bouden et al. [34] developed a system that provided users with a geosimulation functionality, allowing them to manipulate outbreak model parameters such as climate to aid decision makers in visualizing the progression of infectious disease distribution. However, the differences among recently developed systems is not well understood, including the use of development techniques, data sources and evaluation procedures.

A prominent complaint in literature reviews of SDSS is the inconsistency of end user involvement [35]. This is problematic in many respects because there are many considerations for software design, such as available technology and display capability, training, and knowledge of the decision-making process. While the potential and intention behind the design of many decision support systems is laudable, previous research has found these principles are inconsistently applied in the design of SDSS and related surveillance tools targeting public health users [36].

An attractive aspect of SDSS are their integrative capability to draw on many different sources of data, to relieve much of the organizational burden from the end user. However, inclusion of data sources for a given task are becoming increasingly difficult to address the full range of potential contributing factors using established methods. For surveillance tasks and analysis, common methods employ manually curated data that is processed using GIS and statistical software. As surveillance has become increasingly multidisciplinary, there is demand for access to more data and analytical tools [37]. As such, the online community has begun facilitating the acquisition of various types of data for analytical purposes by creating online repositories of data with various states of organization. Integrating these types of sources is imperative to fully explore all contributing factors to zoonotic disease. Additionally, recent SDSS have allowed for automated integration of data collection from multiple relevant sources such as hospitals, laboratories and physician reporting.

In this systematic review, we differentiate the characteristics of recently developed SDSS designed to aid public health practitioners in the identification of high risk areas for zoonotic disease outbreaks. While other literature has summarized the various forms of visualization and analytic tools targeting zoonotic disease, focusing on SDSS development allows for a better understanding of an emerging type of tool that has the potential to better integrate data for modeling and analytical processes to aid decision making. This is important as public health officials continue to rely on more sources with the rise of new online databases and newsfeeds for surveillance, thus increasing the complexity and cognitive load.

The objectives of this systematic review were to:

- To identify and describe current spatial decision support systems developed for identifying zoonotic disease outbreaks in the public health sector.
- To identify the underlying modeling techniques and predictors used in the development of the identified spatial decision support systems.

## Methods

### Selection of studies

To identify articles covering the recent advances in SDSS, we chose to utilize the guidelines for systematic reviews provided by the PRISMA statement [38]. To identify potential articles, we searched the Google Scholar database using Boolean logic to combine key terms described in Table 1 and limited the years of observation from January 1, 2008 to August 31, 2018. The search was performed independently by two researchers, using the Endnote reference manager to document all citations. Once duplicates were removed, titles and abstracts were screened, and discrepancies were resolved through mutual agreement. A final screening of full manuscripts was carried out, whereby inclusion and exclusion criteria were used to identify the final set of articles by two reviewers.

**Table 1:** Columns A, B and C indicate interchangeable terms combined using AND/OR

Column A	Column B	Column C
Spatial decision support systems	Public health	High risk areas
Spatial online platforms	Zoonotic disease	Outbreak detection
Mapping tool	Infectious disease	Cluster detection

Example search terms: (spatial online platforms) and (zoonotic disease) and (outbreak detection)

### Inclusion and exclusion criteria

For a prospective article to be considered, the subject of the title and abstract had to describe the development and implementation of an SDSS designed to aid the decision-making process to manage a zoonotic disease outbreaks capable of spatial modeling. However, we did consider the article if it described a tool that was developed to address reportable infectious disease generically and was inclusive of zoonotic diseases. The main intervention the SDSS had to address was the identification of high risk regions for disease transmission or outbreaks. As the primary concern of this article is to enhance the current understanding of tools available to support public health decision making, we only included those articles that studied human or animal populations. We excluded any summaries or reviews written about current SDSS, in addition to articles which proposed SDSS frameworks yet to be implemented. Any articles that did not develop an SDSS in which the intended users were professionals such as epidemiologists, veterinarians, wildlife biologists, or other similar positions were excluded. Any SDSS that were developed to manage other health conditions exclusively such as cardiac disease, diabetes, obesity, or emergency care were not considered. We also excluded the article if it described a system that did not include fully integrated spatial visualization and modeling capabilities through a user interface. Articles which described tools developed specifically for mathematical modeling of outbreaks or other events alone, and not in conjunction with decision-aid functionality for public health professionals were excluded. Systems which were developed to monitor and maintain a database of current zoonotic disease reports were also not considered. A full description of all criteria are summarized in Table 2.

**Table 2:** Inclusion and exclusion screening criteria

Inclusion criteria	Exclusion criteria
System targets zoonotic diseases or infectious diseases generically, but inclusive of zoonosis	Systematic/scoping review of surveillance systems
Performance of spatial modeling	Does not address decision support for public health officials
Intervention is to identify at risk areas for outbreaks	Non-implemented system, modeling only
Human and animal health only	No user interface with spatial visualization built into system
	Database of zoonotic or infectious disease
	Monitoring system for disease cases only

### Assessment

**Fig. 2:**
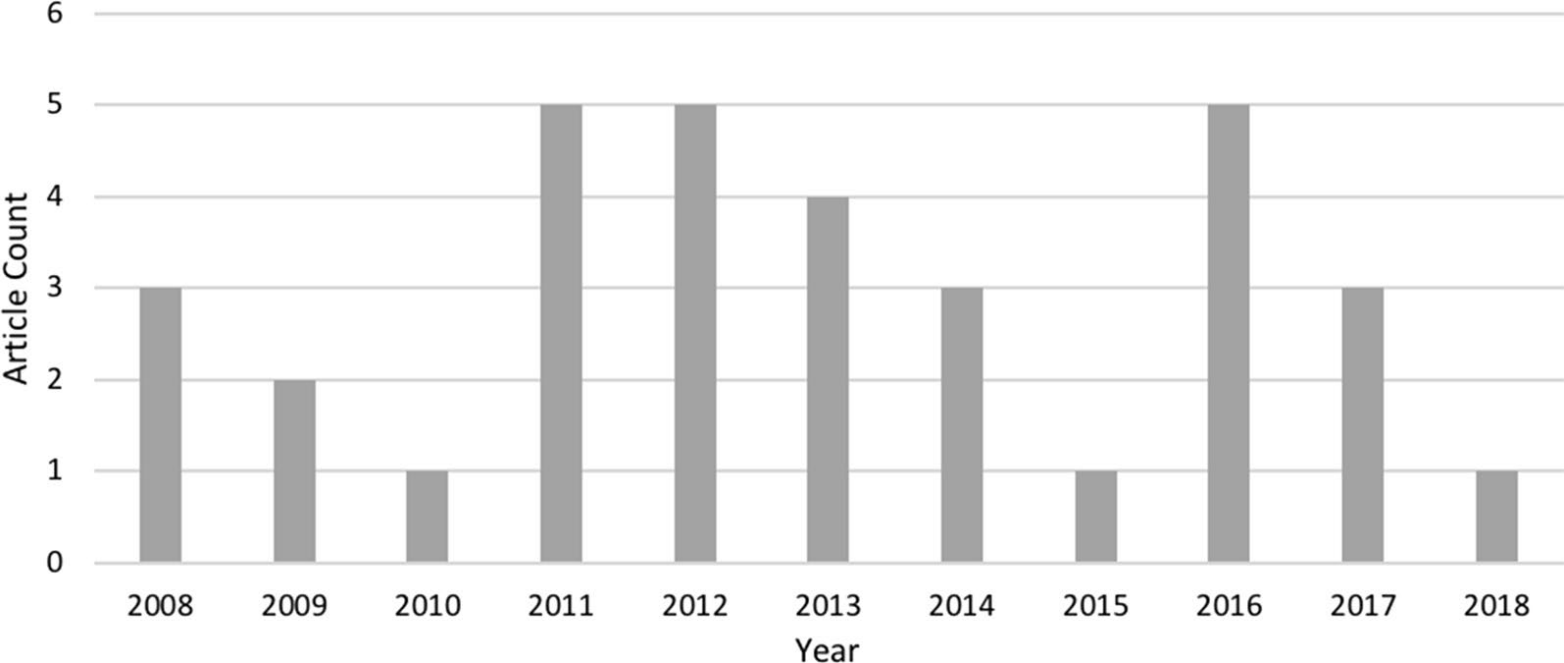
Distribution of studies selected for full text evaluation by publication year

For each article that met our inclusion criteria, we reviewed the entire text in addition to tables, figures and supplementary material. For the review process, we first evaluated the quality of each publication using a scoring methodology (MMAT) approved for use in systematic reviews. This tool first applies screening criteria for any type of study to confirm whether there are clear objectives and whether the collected data allowed the researcher(s) to address the objective. Studies which passed the initial screening question were then further evaluated based on study type (quantitative, qualitative, or mixed methods) using a ranking system to answer specific questions (see Fig. 2), with either: *yes, no, unable to tell,* or *not applicable* similarly to Fournet et al. [39] (each response was ranked as 4, 3, 2, 1 respectively). The Template for intervention description and replication (TIDieR) checklist was then used to evaluate each of the full texts that passed the MMAT screening for completeness in addressing the intervention proposed as a means of qualitative synthesis [40]. The TIDieR was published in 2014 to extend the CONSORT and SPIRIT statements, and can be applied to all evaluation study designs [41]. This checklist covers topics including naming the specific intervention, why, what, who, how, where, and when.

General descriptions of the final set of articles are documented in excel, including the target disease or diseases, the target population at risk, and the geographic coverage, and data sources and types used for identifying areas at risk for outbreak. To compare each SDSS in terms of modeling capability, we documented and described eight necessary characteristics of a SDSS as identified by Sugumaran et al. [29]. These include spatial data management/analysis, visualization, report generations, interactive problem solving, spatial modeling, semi structured problem solving, scenarios evaluation, and easy user interaction.

## Results

### Screening results

**Fig. 3:**
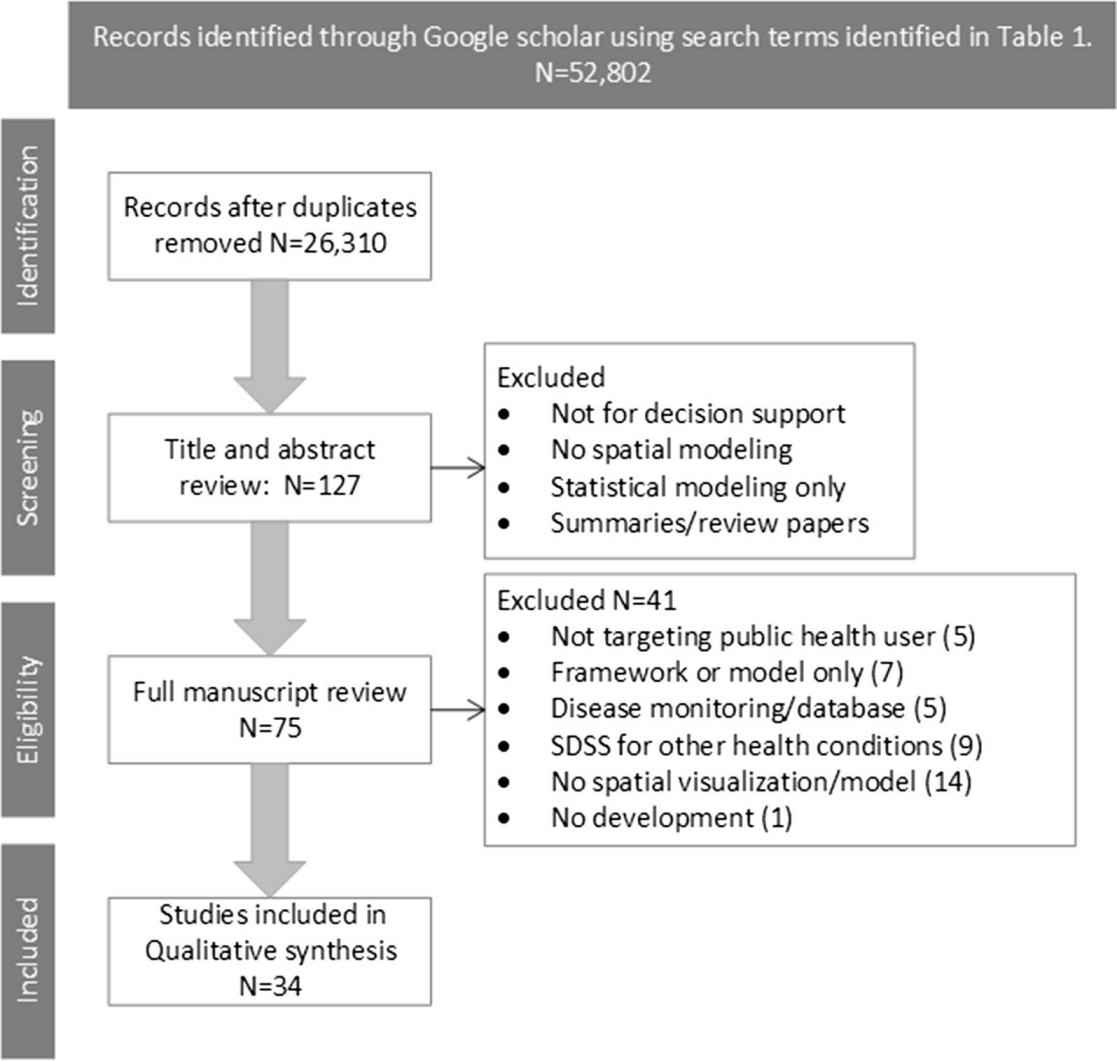
PRISMA diagram featuring article selection and screening process. All papers were selected based on inclusion and exclusion criteria described in Table 2, a detailed explanation of papers excluded with reasons are provided in Additional file 1

After duplicates were removed from search results and inclusion/exclusion criteria were applied to title and abstract screening, 75 articles were subject to full text review. We then screened each full article for inclusion/exclusion criteria and reduced the final set to 34 articles. Of those remaining, all articles selected described SDSS development intended for public health officials as end users which focused on identifying at risk areas of infectious disease outbreaks. The full screening process is summarized in Fig. 3. The final set of articles were published evenly throughout the selected time-period of 2008–2018, with a decreasing trend towards the end of the study period. See Additional file 1 for a basic description of all articles [8, 23, 24, 31–34, 42–109] that underwent full text review, with reasons for exclusion.

### Qualitative assessment

#### Quality filtering

**Fig. 4:**
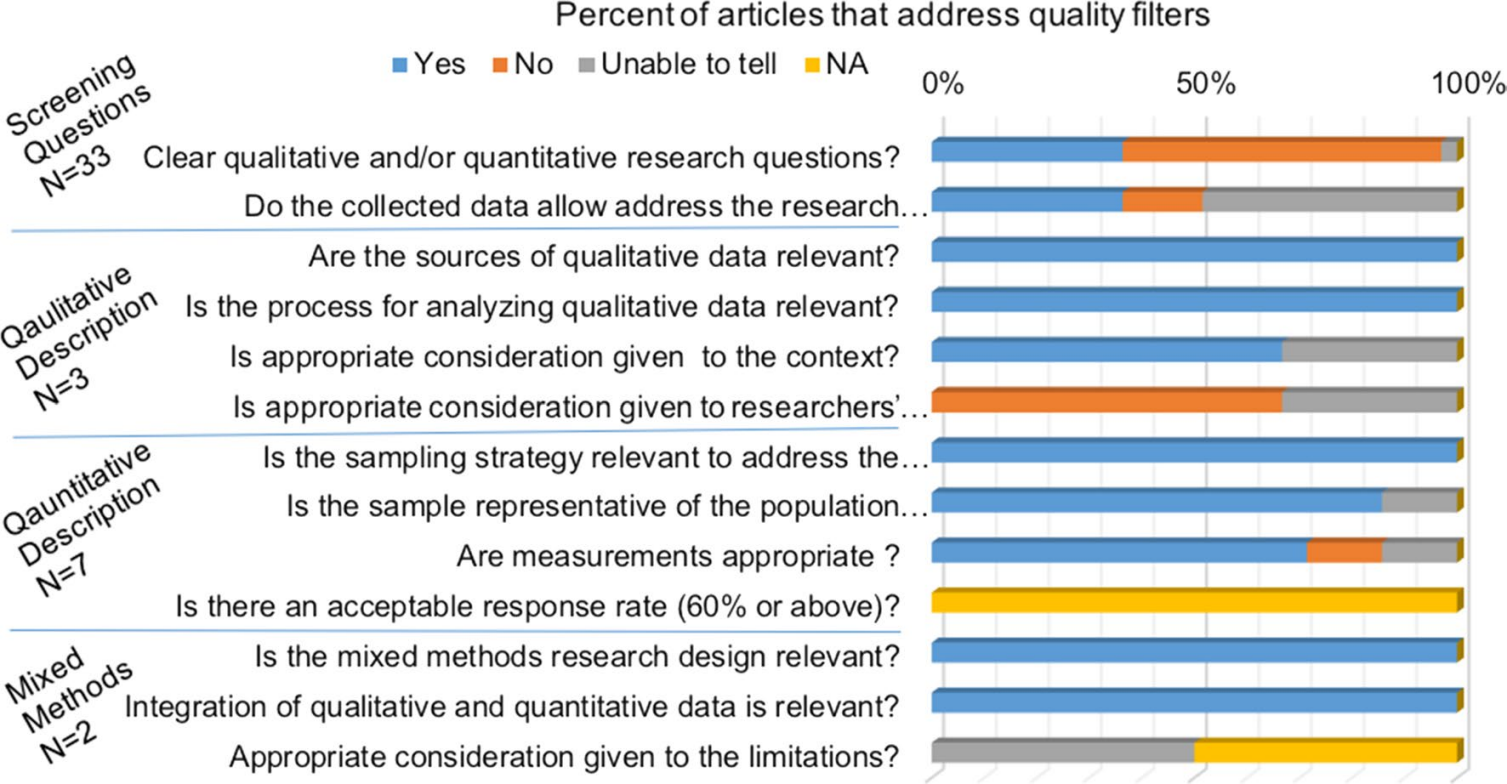
Results of qualitative assessment using the MMAT tool. All papers included in the full text review were subject to the MMAT review, 22 of the 34 papers did not pass the initial screening questions and were not subject to further evaluation

Of the final 34 articles identified after full text screening, 22 did not meet the MMAT screening criteria by addressing a specific objective or research question that could be readily quantitatively or qualitatively assessed (see Additional file 2 and Fig. 4). The articles which could not be fully evaluated using the MMAT generally reviewed the development and implementation of an SDSS, while a few also described a case study (n = 4), a simulation study (n = 3), and a pilot study (n = 1). Of those studies fully evaluated using the MMAT (n = 12), three were qualitative, seven were quantitative, and 2 were mixed methodologies. The average MMAT score for the final set of 12 articles was 87.5%.

#### Quality of interventions description

**Fig. 5:**
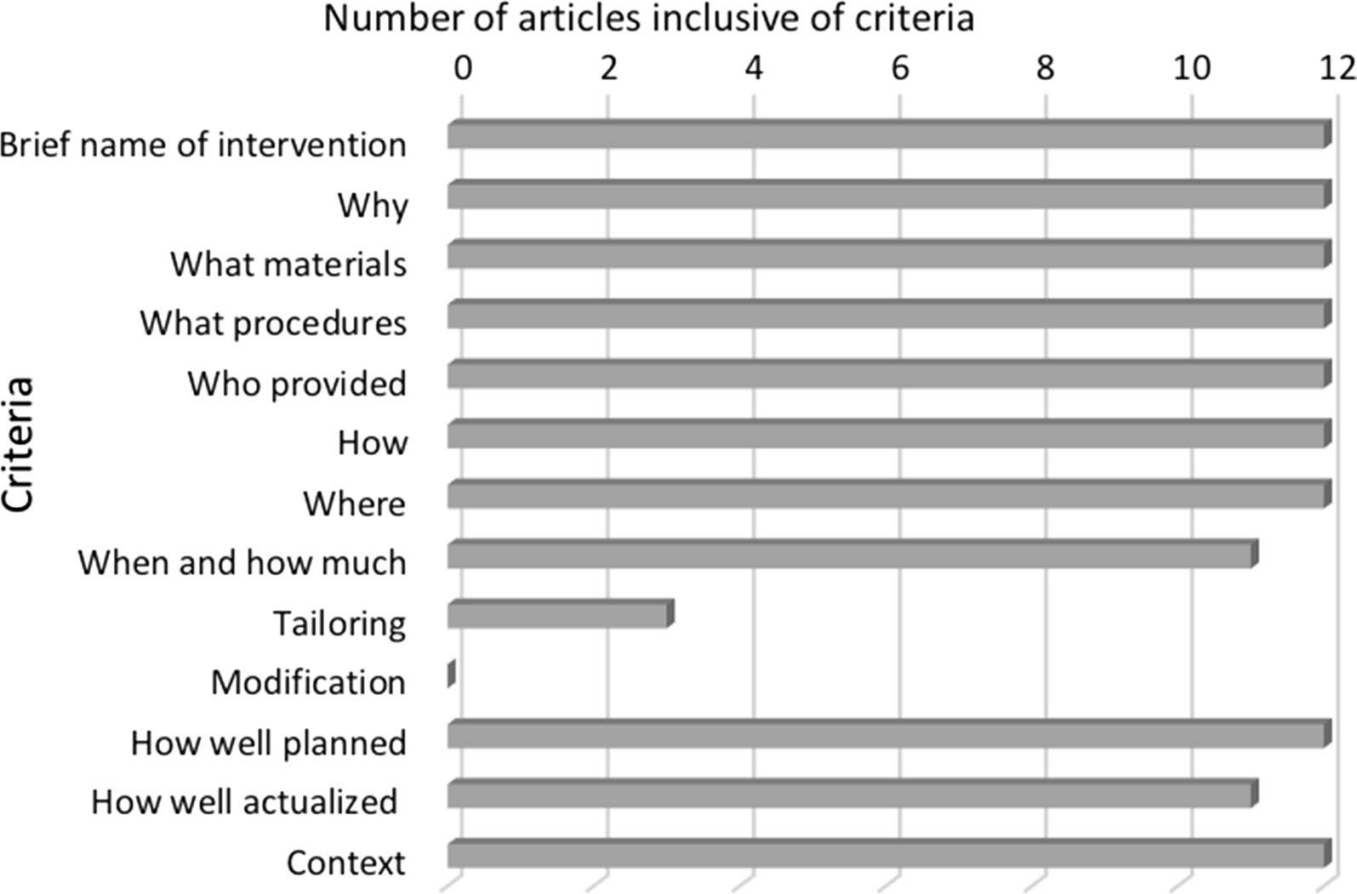
TIDieR checklist criteria results

The papers that passed the MMAT screening questions were also assessed using the TIDieR checklist to determine if the proposed intervention was adequately described (see Fig. 5 and Additional file 3). All studies addressed most items on the checklist, all with a similar intervention to develop a spatial decision support system, though with some variations to address high risk areas for outbreaks and methodology. Rationales behind development varied from visualization of space time events, detection, monitoring or prediction of disease clusters or epicenters, or more specific tasks related to targeting regions for disease elimination or management. All but one [73] article described the intervention as effective, either through sensitivity (n = 3), significance (n = 3), or percentage (n = 2) evaluations on ability to identify at risk areas for outbreaks, in addition to user access traffic (n = 2), and user feedback on usability or usefulness (n = 5).

### Evaluation of selected SDSS

#### General descriptions

The selected SDSS are described briefly in Table 3 (also see Additional file 4). SDSS were developed predominantly as desktop applications, while four were web-based and one was undetermined. Half of the systems were capable of real or near real time surveillance data (n = 6) while the remaining systems were retrospective. Common practices described during development included data preprocessing, cleaning, or aggregation. Aggregation was often applied to climactic or environmental predictors for which finer geographic resolution was available, compared to other data sources. Other difficulties in combining data included differences in storage format, naming conventions, or data relationships from various sources and health organizations. Many studies described methods to address integration issues, though most did not give specifics.

**Table 3:** Articles selected for inclusion for systematic review

Article number	First author	Tool	Year	Intervention
1	Ali	ID-Viewer	2016	Development of visual analytics decision support system for data acquisition, analysis, and visualization for surveillance tasks
2	Bui	Unnamed online analytical tool	2016	Development of web-based integrated system for malaria surveillance
3	Carney	DYCAST	2011	Development of early warning system for West Nile virus outbreaks
4	Chen	Unnamed online analytical tool	2016	Development of online platform to monitor dengue fever
5	Delmelle	H.EL.P.	2011	Development of decision support system for practitioners to understand disease dynamics
6	Gesteland	EpiCanvas	2012	Development of interactive visualization system for disease surveillance
7	Guo	OSCAR	2017	Development of framework to integrate spatial analysis, and data aggregation
8	Iannetti	SIMAN	2014	Integrated web support system in veterinary epidemic emergencies
9	Kelly	SDSS	2013	Development of a surveillance response system for Malaria elimination
10	Rao	SEARUMS	2008	Development of modeling tool to study avian influenza outbreaks, for scenario analysis and visualization
11	Vanmeule-brouke	HIV/AIDS tool	2008	A system to explore hypothesis testing though data integration and visualization to manage HIV/AIDS
12	Wangdi	SDSS for malaria elimination	2016	Development of spatial decision support system to aid malaria elimination

Article numbers assigned refer to the associated SDSS, and will be used to refer to specific papers in later tables

#### Target disease, population, and geographic coverage

Further examination revealed an array of individual or groups of diseases targeted by the selected studies. Most commonly, a system was developed with the intent of being used for monitoring a single disease (n = 7), while those remaining were inclusive of several reportable infectious diseases (n = 5) for a given study area. Many of the reviewed SDSS were developed such that the international (n = 2) community could potentially utilize them, or a specific country (n = 7). A smaller proportion (n = 3) focused on a specific region, state or province. All but one of the reviewed systems focused on human centric health outcomes (Table 4).

**Table 4:** Targeted diseases for selected SDSS

Article	1	2	3	4	5	6	7	8	9	10	11	12
Disease targeted												
Infectious disease	✓				✓	✓	✓					
Malaria		✓							✓			✓
West Nile virus			✓									
AIDS/HIV											✓	
Animal disease								✓				
Dengue virus				✓								
Influenza										✓		
Target population												
Human	✓	✓	✓	✓	✓	✓	✓		✓	✓	✓	✓
Animal								✓				
Geographic region												
International							✓			✓		
Country	✓	✓		✓	✓			✓			✓	✓
Regional						✓			✓			
State or province			✓									

#### Data types and sources

We found that most systems relied on case related data (n = 9) and most frequently was provided by National health departments (n = 7). Case reporting was sometimes combined with other data types (n = 4), though nearly half (n = 5) utilized case reporting data alone. Overall, combining multiple data sources was slightly more common (n = 7). In several instances we found that SDSS often integrated individual reporting, climactic, environmental, or remote sensing data. Less common data sources included drug sales [43], citizen reporting and social media data [57, 64]. A small subset allowed the user to upload data into the system (n = 2), and several SDSS allowed near real time automated data integration with local or national health agencies. One employed web crawling services to automatically update data in near real time from public online databases or resources [64] (Table 5).

**Table 5:** Data types and sources utilized in the described SDSS

Article	1	2	3	4	5	6	7	8	9	10	11	12
Data type												
Confirmed case reports (human)	✓	✓	✓	✓	✓	✓	✓		✓			✓
Confirmed case reports (avian)								✓		✓		
Animal distribution								✓		✓		
Drug sales	✓											
ED chief complaint	✓											
Animal mortality			✓									
Sanitation amenities											✓	
Temperature	✓											
Rainfall	✓											
Environmental data	✓						✓					
Citizen notification			✓									
Human population										✓		
Animal population										✓		
Remote sensing							✓					
Demographic data				✓			✓			✓	✓	
Data sources												
Local hospitals	✓				✓	✓						
Local pharmacies	✓											
Local health department			✓									✓
National health department		✓		✓			✓	✓	✓	✓		✓
International health agency									✓			
Census reporting				✓						✓	✓	
Satellite imagery	✓											
Social Media							✓					
Citizen reporting			✓									
Open source database												
News reports							✓					
User input											✓	✓

### Summary of core SDSS characteristics

#### Spatial database management

An overview of results for the review process in which each SDSS was evaluated based on the inclusion of the eight core functionalities are presented in Table 7. For the first core functionality, all systems described the inclusion of *spatial database management,* however the specific system could not be identified in four instances. ArcGIS and PostgreSQL were the most frequently used management system, open source GIS software such as QGIS while others utilized database management software such as MySQL in combination with map visualization tools such as OpenLayers and Google Maps.

#### Visualization

All systems were built as an application with integrated mapping capability that was developed for user interaction. All SDSS provided similar layouts that included a menu bar at the top, in addition to panels and or tabs that could be navigated to produce different displays including maps, analysis parameters, results, file management, analysis, or summaries. Most base maps were choropleth in nature and allowed overlays or color themes that identified areas of interest. These overlays were often points or circles to draw the user’s attention to georeferenced outbreak data. Other raster format layers representing demographic data, landuse, or climactic data were common. Less common overlays included simulations of networks of disease spread.

#### Report generation

While all SDSS were capable of displaying maps, most applications provided a means to generate tables or charts with summaries of the data or analyses being conducted. Often reports were available in a separate panel or tab within the user interface. A variety of graphs were used, ranging from pie charts for illustrating concepts such as proportions of case reports by region, tables summarizing analysis results, to bar or line graphs often used to present time-series data. Those SDSS which followed a web application format often took advantage of services such as HighCharts, which allow the developer to produce interactive charts within webpages.

#### Interactive problem solving

A variety of built in functions were noted for all SDSS related to mapping, analysis, and graphing. Often the interface allowed the user to adjust or remove layers to customize the spatial visualization in addition to zooming, color themes, legend display, select region etc. When the user performs an analysis, SDSS commonly allowed user selection of species or disease (if more than one available), timeframe, region of interest, model parameters, covariates to include, or graphical output format. In a few instances, an SDSS was capable of several different types of outbreak detection and prediction analysis [43, 48, 83], or allowed for simulations of disease outbreaks [34].

#### Spatial modeling capability

Most commonly choropleth maps were produced for model visualization, often identifying areas in which outbreaks of disease were identified or predicted. Risk maps were not always conceptualized in the same way ranging from clustering, disease activity levels, at-risk households to population density. Many SDSS were capable of also modeling disease distribution, identifying locations of individual cases, or zones of high risk. In one instance transmission routes or disease origins and destinations over a given time frame were modeled within a network to identify high risk nodes [33, 73]. Several spatial and non-spatial analytical techniques were used to develop the set of SDSS, often allowing the user to choose between multiple options including spatial scan statistics, data mining algorithms, susceptible, infected, recovered (SIR) models, Moran’s I, Knox test, and linear regression (see Table 6 for a summary).

**Table 6:** Summary of visual and statistical modeling techniques utilized by selected SDSS

Article	1	2	3	4	5	6	7	8	9	10	11	12
Spatial visualization modeling												
Buffered zones				✓					✓			✓
Point or polygon overlay	✓	✓	✓		✓	✓	✓	✓	✓	✓		
Choropleth maps	✓	✓	✓	✓	✓	✓	✓	✓	✓	✓	✓	✓
Heat/density/risk maps	✓	✓	✓		✓							
Spatial statistics modeling												
Spatial scan	✓	✓	✓	✓								
Spatial autocorrelation algorithms		✓					✓					
Space–time k-function		✓			✓							
Knox test			✓		✓							
Network modeling								✓				
k-nearest neighbor	✓	✓										
Non-spatial statistics modeling												
Correlation						✓						
Anomaly detection									✓			
Support vector machine	✓											
Bayesian model							✓✓					
SIR model										✓		
Regression	✓						✓					

#### Semi structured problem solving

Not all end users would necessarily utilize the same methods in designing a particular SDSS, apart from analyzing outbreaks, as such most SDSS allowed the user to control the analysis and modeling process in several ways. The user could often tune parameters used in the modeling process such as timeframe in which the model was constructed, search radius, predictors, or data sources. Furthermore, some studies allowed the user to query, add to, or modify the database [24, 80, 88]. Additionally, these SDSS often allowed the user to select from different map layers, or graphics to model an outbreak situation to their specifications and could be manipulated by zooming, panning, or feature section.

#### Scenario evaluation

A lot of the same features mentioned for semi structured problem solving also allow the user to iteratively model an outbreak situation to the users’ satisfaction. This is evident through iterative modeling for a given scenario using different data sources, parameters, or map specifications. A small set of studies incorporated methods to compare different models, as Ali et al. [43] allowed the user to compare two evaluations for a given outbreak side by side, which utilized different predictors or chart predicted versus observed outbreak data. Another study provided a feature to simulate the results of different mosquito spraying interventions to prevent diseases such as West Nile virus [34].

#### Easy user interface

All systems provided a graphical user interface that integrated mapping for spatial modeling visualization, and options to produce a variety of graphs or tables to aid the decision-making process either in a desktop or web application format. However, there were few studies that included a mechanism to assess user satisfaction to determine ease of use. A total of five studies included details of a usability evaluation and discussion. Evaluations were carried out by interviewing or surveying the user after performing set tasks. Surveys were either custom made or an industry standard such as the System Usability Scale [110]. Less direct methods of measuring ease of use included tracking user system accesses and usage, and percentage of target user utilization. Several studies indicated usability was addressed during the design process by consulting with potential users on needs and requirements, though follow up evaluation was inconsistent.

Table 7 describes the major characteristics of SDSS’ evaluated here, by summarizing the

**Table 7:** Summary of finding for SDSS core functionalities

Characteristic	Description	Most common method	Less common methods
Spatial data management	GIS based management systems that can organize and analyze spatial data	Unspecified (n = 4)	ArcGIS, Google maps, OpenLayers, QGIS, PostgreSQL, hBase, MySQL
Visualization	Visualization through maps, graphs, tables	Choropleth (n = 12)	Point layers, count overlays, panels, buttons, data entry fields, menu
Reports	Summary of scenario or analytical process, may be graphical, maps etc.	Mapping (n = 12)	Map, table, chart, statistic summary, network graph
Interactive problem solving	Environment which allows the user to explore the possible solution space for a given problem, allowing interaction within the problem-solving environment	Select area of study(n = 6)	Select species, timeframe, covariates, color theme, graph views, radius selection, query fields
Spatial modeling capability	Availability of spatial/non-spatial modeling packages	Clustering(n = 5)	Clustering, risk mapping, anomaly mapping, disease spatial distribution, networks of disease
Semi structured problem solving	Problems that are ill defined, but can accommodate imposed restrictions and user preferences	Adjust analysis parameters (n = 12)	Explore disease network, choose summary statistic, user selection of model parameters
Scenario evaluation	Decision support utilities that allow scenario analyses through iterative analyses	Adjust distribution display (n = 12)	Track different species, transmission route or outbreak simulation, generation of actionable suggestion, distribution of cases/clusters
Easy User interface	Interfaces that engage the user, and allow easy interaction	Usability testing (n = 5)	Design consultation, mental mapping of tasks, feedback surveys, pilot study, usability, and usefulness testing

most common method by which each characteristic is incorporated, and possible formats, or methods.

## Discussion

### General features

In our review, we found a large amount of diversity in design and scope regarding study areas targeted by recent SDSS. The systems observed here were developed by smaller research teams at individual institutions, as well as by government divisions throughout the world to address the concerning zoonotic disease developments and spread which have increased in recent years. Several SDSS built for smaller regions relied on local data sources and reporting, whereas those developed to be more generalizable used more public data sources. These findings highlight a common problem wherein systems are developed in isolation and rely on locally collected and curated data.

Fortunately, the trend in the data presented here indicates most systems are developed to address numerous diseases over large regions, entire countries, or the entire globe. Those systems that are more geographically inclusive have the potential to address a range of decision making needs of public health officials who are often faced with a variety of outbreak threats from surrounding environments. This universal approach reduces the necessity to have multiple tools that perform similar or redundant tasks.

### Core SDSS functionalities

For each of the core functionalities, studies often provided enough detail to determine specific details regarding architectural design that allow visualization, reporting, problem solving etc. and implementation. Throughout the history of SDSS development, there has been a heavy reliance on proprietary, standalone GIS [111]. Beginning with open source GIS distributions such as GRASSGIS, the last 10–15 years have seen a remarkable expansion in the availability of open source software such as statistical packages, web mapping services, and others which have shifted the focus on client–server architecture to a web services model in newer SDSS [111]. The results here indicate that the availability of new tools to aid SDSS development are diversifying what software is used to produce the final product, ranging from desktop applications integrating one of many GIS with spatial database management to the implementation of web application frameworks that utilize open source database management systems (PostgreSQL, MySQL, etc.) with mapping services such as OpenLayers. Also reflected are a large variety of analytical tools or algorithms that can be packaged for use in a variety of systems for a similar purpose, or complementary analysis. For instance, Kulldorff’s spatial scan statistic software [112], k-nearest neighbor and support-vector machine learning models were implemented in just one SDSS reviewed here.

Many of the reviewed studies were less consistent in describing or performing model evaluations, obtaining stakeholder requirements, pilot studies, or usability testing. Several studies described their system as user friendly [23, 48, 49, 51, 64, 106, 107, 113], though not all performed an evaluation or even approached the topic in the same way. Most studies utilizing this term referred to usability testing, which was often followed with robust evaluation methods. However, other studies used the term in reference to ease of configuration with other software, or visual displays that were esthetically appealing without providing details of user participation to evaluate these claims. Despite this, it is noteworthy that usability metrics mentioned within the selected articles do not necessarily evaluate all aspects that potentially contribute to ease of use.

### Current data sources and future developments

Integration of disparate data sources has been a common theme in surveillance systems and decision support systems for public health surveillance [114]. To accomplish this, collections from local health practitioners, state departments, or government agencies may be joined such that reportable disease data are housed within the same framework that integrates sources such as media, genetic sequencing, socio-economic data, or other environmental characteristics. However, integration of multiple data sources was not universal in recently developed SDSS reviewed here. Availability and utilization of social media, news, landscape, climactic, socio-economic data are reflected here, as several systems covered are inclusive of these increasingly readily available data types, providing even more data to better understand what contributes to disease spread and outbreaks. There are still many inequities in accessibility to resources globally that make collection and availability of data difficult, stratifying health status and potential interventions by socio-economic means and access to urban areas [115]. Limited resources to collect and organize data to develop effective surveillance systems is further impacted with difficulties in combining datasets recorded for varying formats, quality standards, and reporting requirements [116].

Genetic sequencing and related bioinformatics tools and resources are an emerging data source which are not well represented in recent SDSS development. Open source genetic data repositories are increasingly more common, such as GenBank [19] and the IRD [20] which contain an abundance of pathogen sequences and relevant metadata that present opportunities to enhance current practices. Utilization of sequencing to support epidemiological and disease prevention efforts have been demonstrated in recent epidemic events including the Ebola outbreak in west Africa from 2014 to 2015, in which genetic sequencing provided valuable information on the genetic diversity, to estimate how fast the disease spread and predict future transmission through phylogenetic analysis [117]. Likewise, Fraser et al. found that transmission potential estimates derived from genetic sequencing were comparable to traditional epidemiological estimates based clinical attack rates during the H1N1 pandemic of 2009 [118]. While these developments show great potential for the use of bioinformatic tools and resources in public health, the public sector has yet to take full advantage of genetic data driven tools. Despite a substantial amount of literature that documents the value of understanding the genetic variability of disease [119–121], only three potential articles included in the screening process were inclusive of sequencing data but did not pass the inclusion criteria or quality filtering process [53, 89, 95]. Complications for including sequencing data for zoonotic surveillance are largely due to limited resources or laboratory capacity to rapidly sequence emerging infections [121], and a unifying framework from which to integrate the phylogenetic patterns of an infectious agent and potential hosts [122], in addition to drivers of disease.

Understanding the phylogenetics of a disease in context with the spatial distribution, environmental characteristics, and potential hosts, assists in the identification of geographic areas that drive zoonotic disease spread and circulation. Ge et al. [123] compared methodologies for H5N1 avian influenza outbreak identification derived from three different fields including phylogenetics, spatial statistics, and epidemiological analysis of socio-ecological determinants of disease such as human and avian population density, migration routes, railways, and inland water, which found that integrating all three serves to better corroborate observed occurrence and estimate ability to spread. Changes in human population, industrialization, global trade, and travel also play a key role in facilitating the introduction or discovery of novel pathogens throughout the world [124], and phylogenetic analysis facilitates tracing the spread of disease through human interactions [125]. Health agencies are now better able to respond to emerging zoonotic diseases through understanding how changes in the genome of a pathogen impacts disease risk and spread by identifying transmission routes, mutation rates, in conjunction with other epidemiological parameters of interest [3]. Substantial concern for potential pandemics due to transmission of zoonotic disease from animal to human populations such as avian influenza [126] has also prompted integration of animal phylogenetic analysis to better understand interaction and transmission which can aid management and preventative measures [127–129]. The need to develop an integrative approach necessary to understand the emerging spatial patterns of zoonotic disease outbreak and spread has long been recognized, with the advent of movements such as “One Health” which call for a global strategy that is multidisciplinary to address the health of humans, animals, and the ecosystem [130]. Future development should focus on assisting in the ease of access and integration of multiple data sources to achieve this goal.

### Future directions

Data integration and availability still pose several challenges in the development of SDSS for zoonotic disease outbreak management, including variability among available sources such as scale, completeness, and timeliness. Potential data sources range from the microscopic to the global scale, necessitating data transformation and projection for appropriate analysis and visualization. While georeferenced datasets describing environmental and climactic phenomena are more readily available, emerging genetic data sets present some challenges, as location of isolation are generally extracted manually from public records or publications. To address visualization of genetic sequence data, there have been efforts to extract geospatial metadata such as location and host from GenBank records, to ease automation of linking relevant sequence data for spatial modeling of disease [131]. Efforts to model outbreaks within a decision support environment which integrate data collected on different spatial scales need to address automated data extraction and transformation such as aggregation of case reports, host population densities, and locations from which isolates were sequenced for a region under study such that visualization of a multifaceted scenario is possible.

Sparsity in collected data across large geographic regions could also introduce uncertainty in results, leading to imputation or summarization methods to account for unobserved data. This is particularly a problem for genetic sequence data currently available, which does not cover regions comprehensively and geographic metadata may vary by location specificity, ranging from coordinates to country [132]. Machine learning algorithms have been utilized in clinical decision support systems to address missing patient data to aid medical professionals with decision making [133], and may also prove useful in spatial decision support systems to address this limitation. Other approaches to documenting disease case reporting that have been increasingly utilized are social media, new sources and various forms of citizen science. One SDSS reviewed here incorporated public reports of disease morbidity to enhance surveillance, provided through a hotline or online submission. The advent of the mobile phone in an increasingly interconnected digital age has given rise to a form of citizen science aptly described by the phrase ‘Wikification of GIS by the masses’ (WGM) coined by Kamel Boulos [134], and later described as volunteered geographic information (VGI) [135]. WGM is the specific embodiment of crowdsourcing approach in GIS [136]. WGM encompasses data contributions from citizens to bring local knowledge and spatial awareness on matters of interest into better focus. WGM is commonplace now in applications such as Google maps, and similar platforms have arisen to assist in disease surveillance such as Flu Near You [137, 138]. Recent literature has questioned the utility of WGM for surveillance purposes, suggesting that crowd sourced reporting at the local level does not correlate well with CDC records [138]. However, this citizen reporting through crowd sourcing can potentially complement traditional data sources.

Timeliness of emerging genetic data sources are currently primarily used for retrospective study due to inability to perform sequencing locally during epidemiological investigations, especially in regions of the world with limited resources and infrastructure. However, advances in near real time genetic sequencing may address this problem in the near future with the arrival of a portable sequencing instrument, which can produce sequencing results within a day of receiving a sample during on ongoing epidemic [139].

Future SDSS also need to better support existing public health surveillance practices, skills, resources and methodologies [140]. Previous work indicates that LHD’s utilize a wide variety of information systems for surveillance and identifying at risk areas in the United States alone, with little understanding of effectiveness [141]. Incorporating user centered design principles such as designing for the user tasks would allow more effective, efficient work within a user-friendly interface. Sutcliffe et al. has previously described a design framework for visualization decision support tools for epidemiology which incorporates user centered design [101]. They argue that to effectively translate data into actionable policy or prevention measures, the software designers and end-user analysts must collaborate extensively. This approach has been implemented more consistently in clinical informatics projects such as the design of electronic health records [142], though rarely in public health informatics [101]. The successful implementation of decision support tools is often limited to evaluations of the proposed system in which potential users provide valuable feedback, and frequently suffer from user dissatisfaction due to a mismatch in expectation, user knowledge of spatial analysis and other contributing factors [143]. While several SDSS here did evaluate usability after system design, often without prior user input, our finding here indicate that improvements can still be made to better include potential users throughout the design process.

### Limitations

To limit the possible number of comparisons to more dated methods and technology, we focused on more recent work and thus excluded articles prior to 2008. We also did not search all available databases, opting for the breadth of Google scholar which might have resulted in unique publications that were not available in individual databases.

## Conclusions

The characteristics, data sources, development and modeling techniques implemented in the design of recent SDSS that target zoonotic disease outbreak risk were highlighted. To take advantage of data collected on zoonotic diseases and their geographic distribution for public health decision making, it is necessary to integrate current organizational and analytical methods employed into the development of novel systems. These approaches need to be grounded in epidemiological practice, to better serve the end user’s objective. However, the systems described here often did not consistently or effectively address this important aspect. A focus on a cohesive methodology in the development of SDSS is needed to better address the needs of the user. As indicated here, there is a disconnect between many available systems and the end user as many of the recent designs and system evaluations did not include public health officials, or interaction was not limited. Inclusion and better communication with public health officials in the development process is necessary going forward, in addition to ongoing training, planning for full implementation and distribution.

Data integration with new complimentary fields of study are also needed, such as incorporating genetics into zoonotic spatiotemporal surveillance approaches. This enables health agencies to consider not only traditional epidemiological data such as location and timing of reported cases but also the genetics of the virus that influence the virus’s ability to adapt as it spreads and proliferates throughout an environment. Data quality and completeness will also need to be taken into consideration during this process. Going forward with inclusion of new data sources will necessitate an increase in complexity and connectivity between online resources and new public health SDSS in a streamlined manner, to reduce cognitive overload. Few of the SDSS described here have been widely distributed or consistently implemented by the greater public health community. Future systems that adopt these practices will potentially have better support from the target audience, resulting in continuing improvement of SDSS and prolonged use.

## Additional files

**Additional file 1.** Full text screening summary: Full text articles reviewed for inclusion and exclusion criteria.

**Additional file 2.** MMAT assessment: Articles assessed using the MMAT tool.

**Additional file 3.** TIDieR assessment: Articles assessed using the TIDieR tool.

**Additional file 4.** SDSS summarization: Characteristics of final SDSS selection.

## Abbreviations

MERS: Middle Eastern Respiratory Syndrome
WNV: West Nile virus
OIE: Institute for International Epizootics
NGS: Next Generation Sequencing
IRD: Influenza Research Database
GIS: geographic information systems
ESRI: Environmental Systems Research Institute
SDSS: spatial decision support system
CDC: The Centers for Disease Control and Prevention
GADS: geodata analysis and display system
LHD: local health departments
SIR: susceptible, infected, recovered
WGM: Wikification of GIS by the masses
VGI: volunteering geographic information
