# Correction to: A systematic review of spatial decision support systems in public health informatics supporting the identification of high risk areas for zoonotic disease outbreaks

**DOI:** 10.1186/s12942-021-00293-x

**Published:** 2021-08-24

**Authors:** Rachel Beard, Elizabeth Wentz, Matthew Scotch

**Affiliations:** 1 College of Health Solutions, Arizona State University, Phoenix, AZ USA; 2 Center for Environmental Health Engineering, Biodesign Institute, Arizona State University, Tempe, AZ USA; 3 School of Geographical Sciences and Urban Planning, Arizona State University, Tempe, AZ USA

## Correction to: Int J Health Geogr (2018) 17:38 10.1186/s12942-018-0157-5

Following publication of the original article [1], the authors realised that the reference “Iannetti 2014” mentioned in Table 3 was not in the list of References, where ref. #66 is wrongly pointing to a different article from “Iannetti 2011”. The correct Ref. #66 is given below:

66. Iannetti S, Savini L, Palma D, Calistri P, Natale F, Di Lorenzo A, Cerella A, Giovannini A. An integrated web system to support veterinary activities in Italy for the management of information in epidemic emergencies. Prev Vet Med. 2014; 113(4):407–16.
